# *Toxocara canis* and *Toxocara cati* Somatic and Excretory-Secretory Antigens Are Recognised by C-Type Lectin Receptors

**DOI:** 10.3390/pathogens10030321

**Published:** 2021-03-09

**Authors:** Marie-Kristin Raulf, Bernd Lepenies, Christina Strube

**Affiliations:** 1 Institute for Parasitology, Centre for Infection Medicine, University of Veterinary Medicine Hannover, 30559 Hanover, Germany; Marie-Kristin.Raulf@tiho-hannover.de; 2 Institute for Immunology, University of Veterinary Medicine Hannover, 30559 Hanover, Germany; bernd.lepenies@tiho-hannover.de; 3 Research Center for Emerging Infections and Zoonoses, University of Veterinary Medicine Hannover, 30559 Hanover, Germany

**Keywords:** MGL-1, MCL, Dectin-1, PRR, immune evasion, toxocarosis, toxocariasis

## Abstract

*Toxocara canis* and *Toxocara cati*, the worldwide occurring intestinal roundworms of canids and felids, represent an important public health threat due to various disease manifestations in humans. Host recognition of pathogens is mediated by pattern recognition receptors (PRRs). Myeloid C-type lectin receptors (CLRs) are PRRs and recognise carbohydrate structures of various pathogens. As *Toxocara* excretory-secretory products (TES) are predominantly composed of glycoconjugates, they represent suitable targets for CLRs. However, the range of host-derived CLRs recognising *Toxocara* spp. is still unknown. Using a CLR-hFc fusion protein library, *T. canis* and *T. cati* L3 somatic antigens (TSOM) were bound by a variety of CLRs in enzyme-linked immunosorbent assay (ELISA), while their TES products interacted with macrophage galactose-type lectin-1 (MGL-1). Two prominent candidate CLRs, MGL-1 and macrophage C-type lectin (MCL), were selected for further binding studies. Immunofluorescence microscopy revealed binding of MGL-1 to the oral aperture of L3. Immunoblot experiments identified distinct protein fractions representing potential ligands for MGL-1 and MCL. To evaluate how these interactions influence the host immune response, bone marrow-derived dendritic cell (BMDC) assays were performed, showing MCL-dependent *T. cati*-mediated cytokine production. In conclusion, MGL-1 and MCL are promising candidates for immune modulation during *Toxocara* infection, deserving further investigation in the future.

## 1. Introduction

*Toxocara canis* and *Toxocara cati*, the dog and cat roundworm, are worldwide-distributed intestinal helminths with frequent exposure to humans [1]. Humans can act as paratenic hosts by accidental ingestion of embryonated eggs or larvae in tissues of animal paratenic hosts [2]. Human toxocarosis is considered one of the “Neglected Parasitic Infections”, a group of five parasitic diseases that have been targeted by the Centers for Disease Control and Prevention (CDC) as a priority for public health action [3,4]. Since third-stage larvae (L3) are not able to develop further in the paratenic host after somatic migration, they persist in the tissues of, for example, the liver, muscles, eyes or brain. Thereby, they may cause a broad range of clinical symptoms classified into four different forms of toxocarosis—covert toxocarosis as well as the visceral and ocular *larva migrans* syndrome and neurotoxocarosis (NT), with high relevance for human health [5]. Interestingly, migrating and persisting L3 can evade the host immune response, thus persisting for up to a decade within the paratenic host [6].

Generally, parasitic helminths are known for modulating immune responses of definitive and paratenic hosts. Adaptive immunity to *Toxocara* spp. is predominantly characterised by the differentiation of CD4^+^ T helper cells to type 2 subsets (Th2) with the release of type 2 regulatory cytokines such as IL-4, IL-5, IL-10 and IL-13, mediating, for example, the differentiation of B cells (IL-4) and eosinophils (IL-5) [6,7]. These observations are manifested in murine models of toxocarosis, but have also been reported for human patients suffering from different forms of toxocarosis [8,9,10,11,12,13]. Resulting hyperleukocytosis, eosinophilia with infiltration to the site of larval persistence and elevated IgE levels are common hallmarks of helminth infections [9]. Helminths may also initiate innate immunity, for instance by promoting the activation antigen presenting cells (APCs) such as alternatively activated macrophages [14]. However, the role of APCs in *Toxocara* infection is poorly understood. Helminth persistence in definitive and paratenic hosts is also mediated by active immune suppression. In this context, *Toxocara* spp. release soluble antigens called *Toxocara* excretory-secretory products (TES), which influence host immune cells [15].

Initial recognition of pathogens and pathogen-associated molecular patterns (PAMPs) is mediated by pattern recognition receptors (PRRs) expressed on APCs. C-type lectin receptors (CLRs) represent PRRs that predominantly recognise carbohydrate structures of bacteria, viruses, fungi and parasites, often in a Ca^2+^-dependent manner [16]. TES secreted by *Toxocara* spp. L3 contain a large number of glycosylated molecules and proteins like TES-26 (Tc-PEB-1) [17], TES-32 (Tc-CTL-1), TES-70 (Tc-CTL-4) and TES-120 (MUC-1 to 5) [18,19], representing potential targets for CLRs. Interestingly, TES-32 as well as TES-70 represent parasite-derived C-type lectins (PDCTLs) [19,20,21]. These PDCTLs show a significant homology to mammalian-derived myeloid CLRs like the rat serum mannose binding protein A (MBP-A) or macrophage mannose receptor (MRR) [20,21]. Previous studies highlight the role of the CLRs Dendritic Cell-Specific Intercellular adhesion molecule-3-Grabbing Non-integrin (DC-SIGN) and macrophage galactose-type lectin (MGL) in the recognition of *T. canis* TES [22].

However, the range of host-derived CLRs binding *T. canis* TES still remains to be investigated. Furthermore, to the best of our knowledge, nothing is known on CLR recognition of *T. canis* and *T. cati* somatic antigen or *T. cati* TES, and how these interactions influence the host immune response. Therefore, we aimed to investigate CLR binding to *Toxocara* somatic antigens (TSOM) and TES using a comprehensive CLR-hFc fusion protein library, and provide first insights into natural binding properties as well as the immunological relevance of such interaction for the two most promising candidate CLRs.

## 2. Results

### 2.1. CLR Binding to Toxocara spp. TSOM and TES

**Figure 1:**
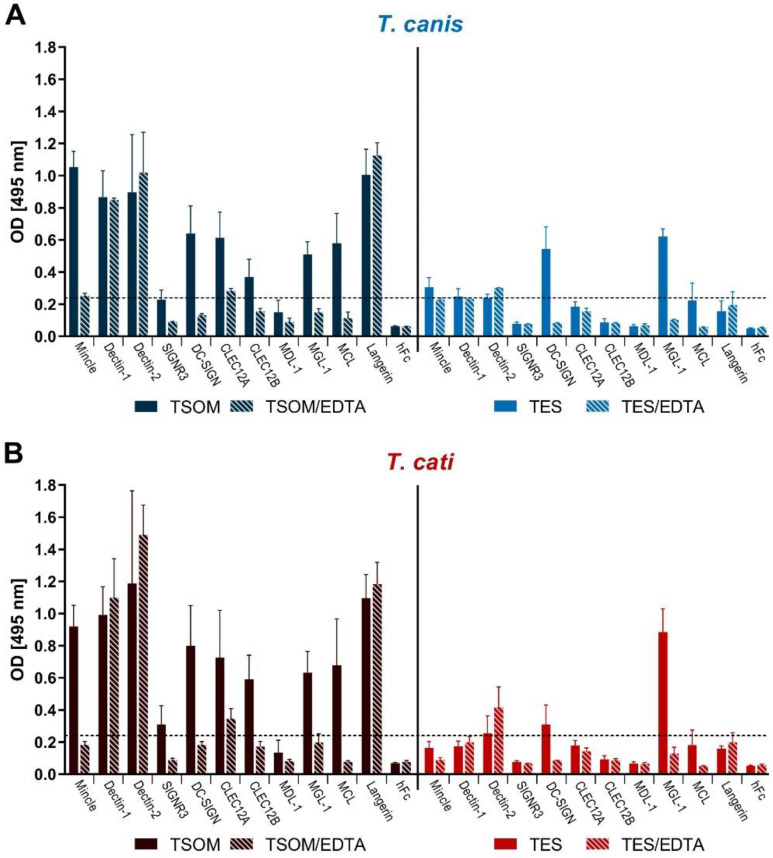
Enzyme-linked immunosorbent assay (ELISA)-based binding studies of *T. canis*- (**A**) and *T. cati*- (**B**) antigens using a murine C-type lectin receptor (CLR)-hFc fusion protein library. Data are displayed as mean + SD. TSOM: *Toxocara* somatic antigen, TES: *Toxocara* excretory-secretory antigen, EDTA: ethylenediamine tetraacetic acid (test for Ca^2+^ dependency), hFc: negative control.

The CLR-hFc enzyme-linked immunosorbent assay (ELISA) was used as a first indicator of direct CLR/*Toxocara* interaction and to identify promising candidates for further tests. CLR-binding was defined as an optical density (OD) value of 0.24 (four times the averaged OD 0.06 of the hFc negative controls). Both, *T. canis* and *T. cati* TSOM were bound by several CLR-hFc fusion proteins such as macrophage inducible C-type lectin (Mincle), Dectin-1, Dectin-2, SIGNR3, DC-SIGN, CLEC12A, CLEC12B, MGL-1, macrophage C-type lectin (MCL) and Langerin, indicating a potential role of these CLRs in recognition of *Toxocara* spp. (Figure 1). Of these CLR/*Toxocara* interactions, Dectin-1, Dectin-2 and Langerin binding was Ca^2+^-independent as indicated by absent reduction in binding by Ca^2+^ complexation using ethylenediamine tetraacetic acid (EDTA)-containing buffer. *p*-values varied between ≤0.001 to 0.500 and are provided in detail in Table S1.

Interestingly, only a limited number of CLRs recognised TES. In particular, MGL-1 substantially bound to both *T. canis*- and *T.cati* TES, while DC-SIGN strongly recognised *T. canis* TES only (Figure 1).

### 2.2. MGL-1 but Not MCL Binds to Toxocara spp. Larvae

**Figure 2:**
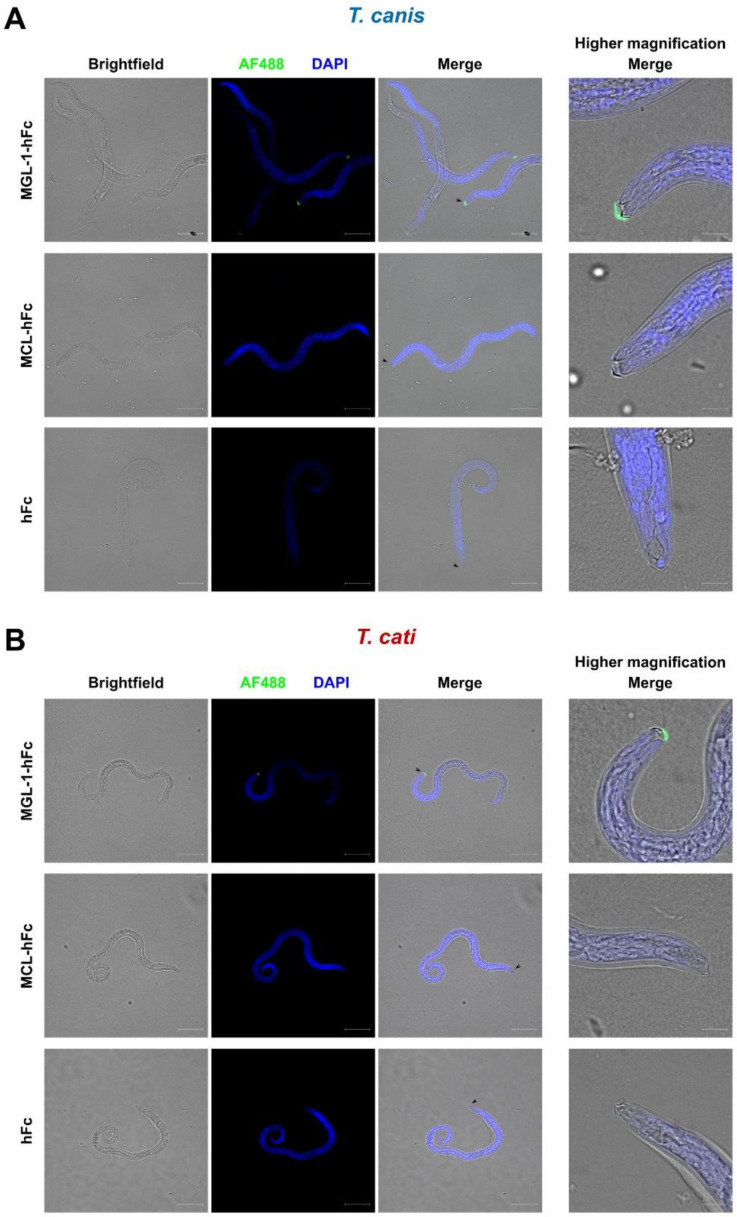
Fluorescence microscopy reveals binding of macrophage galactose-type lectin-1 (MGL-1) but not macrophage C-type lectin (MCL) to the oral aperture of *T. canis* (**A**) and *T. cati* (**B**) L3. Scale bar represents 50 µm for lower magnification (left columns) and 10 µm for higher magnification (right column). Green fluorescence: CLR-hFc fusion protein, blue fluorescence: DAPI-stained DNA, hFc: negative control.

MCL and MGL-1 were chosen as promising candidates for the recognition of *Toxocara* species due to the novelty of MCL/helminth interaction and distinct MGL-1/TES binding (cf. Section 2.1). To test recognition of infective larvae, binding studies involving fluorescence microscopy were performed. Interestingly, only MGL-1-hFc recognised structures present on the surface of *T. canis* and *T. cati* L3, where binding to the oral aperture, mainly to the area of buccal lips and associated surface coat, was observed (Figure 2). Occasionally, a clearly defined sheath composed of the cuticle of the second stage larvae remained during the hatching process. Here, binding of MGL-1-hFc to the same area was visible (Figure S1). Nonspecific binding of the hFc part of fusion proteins to the surface of L3 could be excluded as the hFc control did not exhibit fluorescence (Figure 2). In contrast, no binding signals were visible when *Toxocara* larvae were incubated with MCL-hFc (Figure 2).

### 2.3. Interaction of MGL-1 and MCL with Toxocara TSOM and TES in CLR-hFc Immunoblot

**Figure 3:**
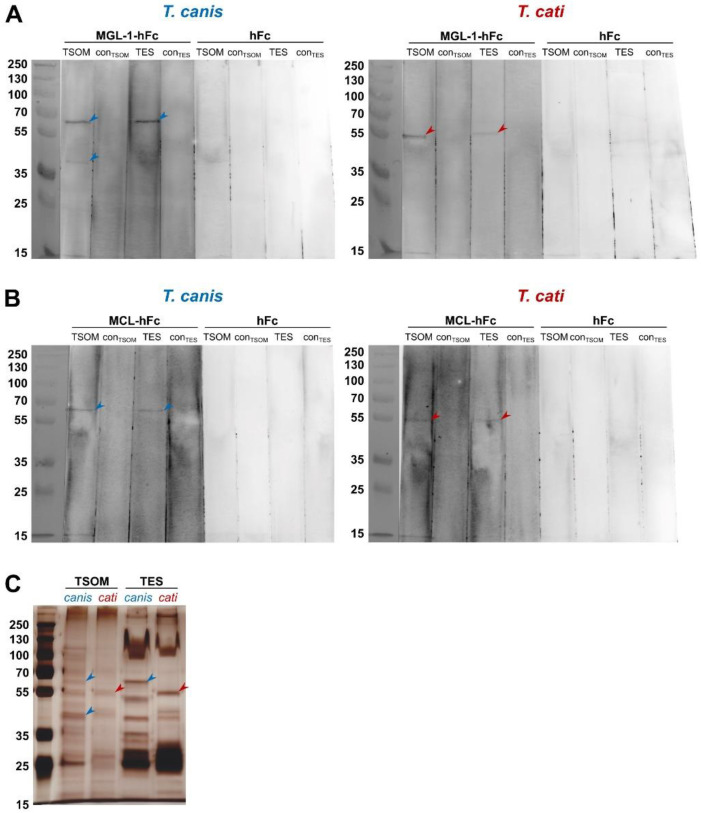
MGL-1 (**A**) and MCL (**B**) recognise distinct *Toxocara* spp.-derived protein fractions in CLR-hFc immunoblot. Silver-stained compounds of *Toxocara* spp. antigens (**C**). TSOM: *Toxocara* somatic antigen, con_TSOM_: phosphate-buffered saline (PBS) control, TES: *Toxocara* excretory-secretory antigen, con_TES_: medium control, hFc: negative control. Note that the membrane had to be dis- and reassembled for differential incubation with the respective fusion proteins and the hFc negative control.

To identify specific protein fractions of TSOM and TES recognised by the candidate CLRs MGL-1 and MCL, CLR-hFc immunoblot was performed. Specific MGL-1 recognition of different components of *T. canis* TSOM was indicated by two bands at approximately 65 kDa and 40 kDa. In case of *T. canis* TES, only one band at approximately 65 kDa was visible (Figure 3A). *T. cati* showed different MGL-1-specific banding patterns. Here, TSOM exhibited only one specific band at approximately 55 kDa. A fraction at this molecular weight was also bound in *T. cati* TES (Figure 3A). In contrast, interaction of MGL-1 with a 15 kDa band in both *T. canis*- and *T. cati* antigens was considered unspecific as faint signals also appeared in the hFc controls.

Interestingly, binding of MCL to *T. canis* and *T. cati* TSOM and TES was comparable to MGL-1 with the exception that no band at the 40 kDa *T. canis* TSOM protein fraction was observed (Figure 3B). Again, unspecific binding to a 15 kDa band in *T. canis*- and *T. cati* antigens was indicated by faint appearance in the hFc controls.

Bands visible in immunoblots corresponded to distinct silver-stained protein fractions of the respective antigens (Figure 3C). Overall, CLR-hFc immunoblot indicated that *Toxocara* spp. ligands for MGL-1 and MCL are (glyco-)proteins.

### 2.4. MGL-1 Does Not Influence Toxocara spp.-Mediated Dendritic Cell Effector Functions

**Figure 4:**
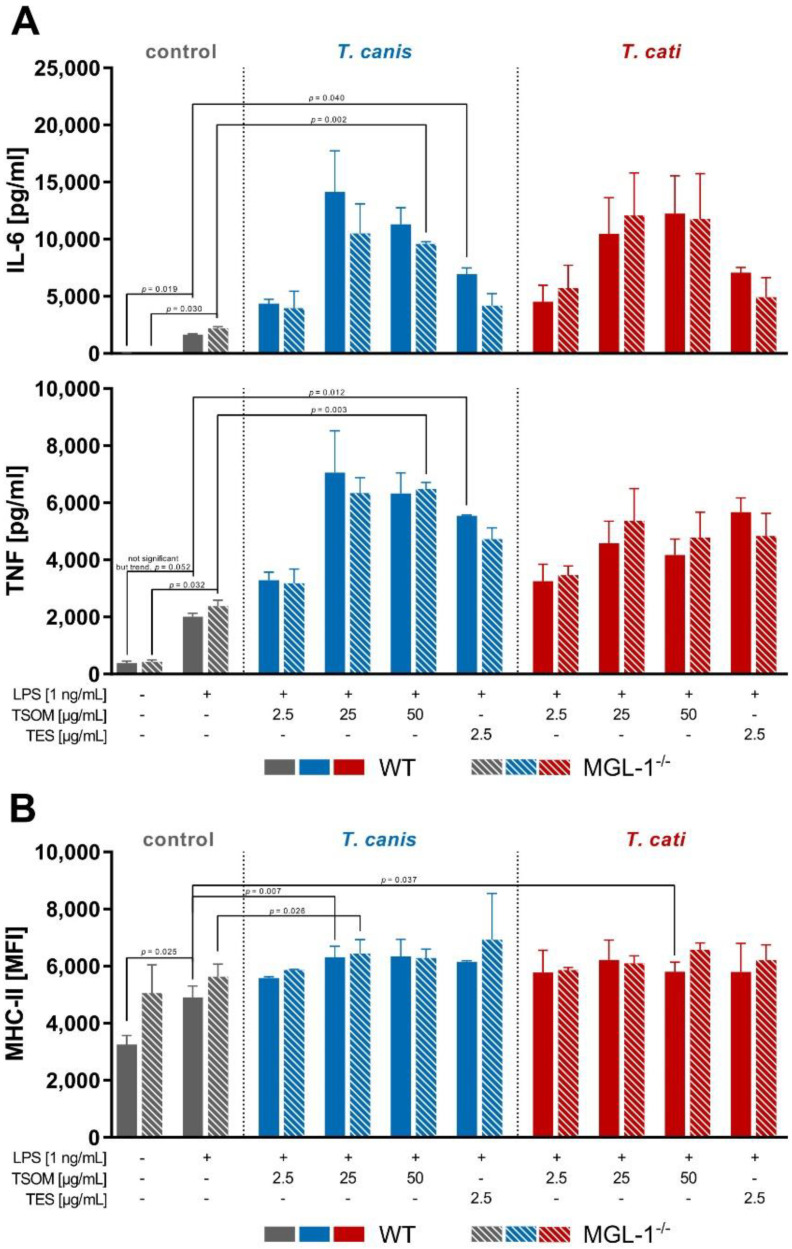
*Toxocara* spp.-mediated cytokine secretion (**A**) and MHC-II surface marker expression (**B**) of wild-type (WT) and MGL-1^−/−^ bone marrow-derived dendritic cells (BMDCs). Graphs are presented as mean + SD of two experiments in duplicates. A *p*-value ≤ 0.05 was considered statistically significant. TSOM: *Toxocara* somatic antigen, TES: *Toxocara* excretory-secretory antigen, LPS: positive control. Note that in this assay co-stimulation of *Toxocara* antigens with LPS was performed due to functional reasons (cf. Section 4.7).

To gain first insights whether MGL-1 might play a role in *Toxocara*-induced APC effector functions, bone marrow-derived dendritic cell (BMDC) stimulation assays were performed. We assessed the Th2-driving cytokine IL-6 and the early pro-inflammatory cytokine tumour necrosis factor (TNF) as these were previously shown to be affected upon *Toxocara* infection or *Toxocara* antigen stimulation [8,10,11,12,23,24,25,26]. In general, stimulation of BMDCs with lipopolysaccharide (LPS) resulted in a significant increase of IL-6 and TNF secretion, which was even more pronounced upon TSOM and TES stimulation (Figure 4A). *Toxocara* stimulation-dependent increase in cytokine secretion compared to LPS was rarely significant, but this is most likely attributable to the high SD due to substantial variation in cytokine secretion of BMDCs derived from different animals.

MHC-II surface marker expression was significantly elevated upon *T. canis* TSOM stimulation of wild-type (WT) and MGL-1^−/−^ BMDCs (Figure 4B). Additionally, *T. cati* TSOM stimulation led to a significant increase in MHC-II expression, which was restricted to WT BMDCs. CD86 surface marker expression also increased upon *Toxocara* spp. antigen stimulation (data not shown).

Overall, observed effects were MGL-1-independent as indicated by the lack of significant differences in cytokine secretion and surface marker expression of MGL-1^−/−^ compared to WT BMDCs. Thus, *Toxocara*/MGL-1 interaction appears to play no role in the assessed BMDC effector function.

### 2.5. Relevance of MCL on Toxocara spp.-Mediated Dendritic Cell Effector Functions

**Figure 5:**
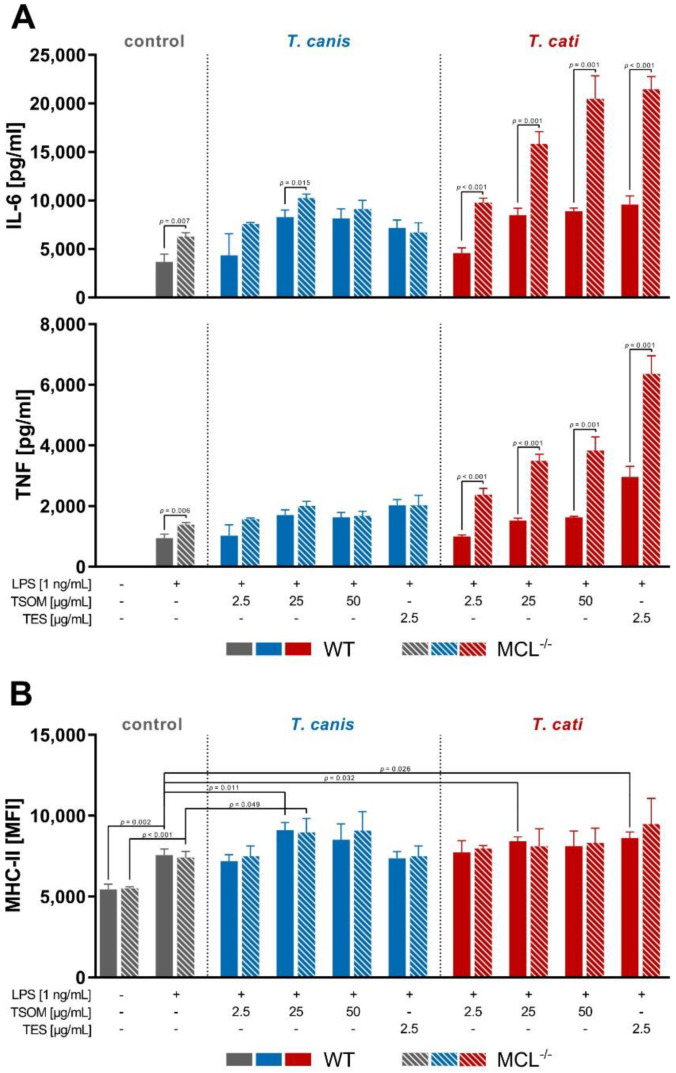
*Toxocara* spp.-mediated cytokine secretion (**A**) and MHC-II surface marker expression (**B**) of WT and MCL^−/−^ bone marrow-derived dendritic cells (BMDCs). Graphs are presented as mean + SD and are representative of three independent experiments in duplicates. A *p*-value ≤ 0.05 was considered statistically significant. TSOM: *Toxocara* somatic antigen, TES: *Toxocara* excretory-secretory antigen, LPS: positive control. Note that in this assay co-stimulation of *Toxocara* antigens with LPS was performed due to functional reasons (cf. Section 4.7).

Analogous to MGL-1^−/−^ BMDC assays, we investigated whether WT and MCL^−/−^ BMDCs show altered effector functions upon LPS/*Toxocara* spp. antigen stimulation. In general, the stimulation of BMDCs with TSOM and TES significantly elevated levels of the pro-inflammatory cytokine TNF and the Th2-driving cytokine IL-6 compared to the LPS control (Figure 5A). Detailed *p*-values of respective *Toxocara* spp. antigen stimulations compared to LPS stimulation are provided in Table S2.

Furthermore, stimulation with *T. canis* TSOM significantly enhanced the expression of the surface molecule MHC-II on both MCL^−/−^ and WT BMDCs, whereas *T. cati* TSOM and TES had a significant effect on WT BMDCs only (Figure 5B). Interestingly, CD86 surface marker expression showed significant differences between WT and MCL^−/−^ BMDCs. However, this effect was also observed in unstimulated cells, pointing towards an intrinsic difference between WT and MCL^−/−^ BMDCs (data not shown).

Strikingly, *T. cati* TSOM- and TES-stimulated MCL^−/−^ BMDCs displayed a significantly increased cytokine secretion of IL-6 and TNF compared to WT BMDCs, indicating that MCL is involved in *T. cati*-mediated secretion of cytokines by DCs (Figure 5A). However, MHC-II expression on the surface of BMDCs was not affected by MCL deficiency.

## 3. Discussion

*Toxocara* spp. are known to modulate host responses to efficiently evade the immune response, thus persisting for long periods within the hosts’ tissues. While most attention has been paid to the adaptive immune response upon infection, little is known about the relevance of the innate immune system mediating initial recognition of pathogenic structures through PRRs in toxocarosis. Immune responses to *Toxocara* infection are predominantly initiated by tissue-dwelling L3 secreting immunologically active TES [6,27]. TES, which is the key driver of *Toxocara*-mediated immune evasion, is mainly composed of glycoconjugates [28]. Host-derived glycan-binding proteins, especially CLRs, recognise those glycans, thus shaping the immune response promoting parasite survival—a process, which is referred to as “glycan gimmickry” [29]. Despite the well-known immunological relevance of carbohydrate structures present in TES [22], almost nothing is known about the recognition of these parasite-derived products by host-derived CLRs. To the best of our knowledge, only binding of human DC-SIGN and human MGL to *T. canis*-derived TES was previously reported [22]. Thus, we aimed to identify promising host-derived CLRs involved in the recognition of *T. canis* and *T. cati*.

As an initial screening, we tested direct binding of CLRs to *Toxocara* spp.-derived antigens by the use of a comprehensive murine CLR-hFc fusion protein library. These CLR-hFc chimeras are established and valuable tools for identification of novel CLR/ligand interactions. For instance, recent studies revealed the recognition of different pathogens such as bacteria and viruses, but also products derived from the protozoan parasite *Plasmodium berghei* ANKA by these fusion proteins [30,31,32]. To obtain a comprehensive picture, ELISA binding studies were not restricted to immunologically active TES but included L3 TSOM as well. The latter was included as it can be assumed that upon larvae death due to potential immunological clearance or aging TSOM is released and subsequently exposed to CLRs present on the surface of APCs. TSOM are composed of a complex mixture of glycostructures present on proteins, lipids, nucleic acids and others [33,34,35], most likely containing ligands for a manifold of CLRs. This was reflected by binding of a variety of CLR-hFc chimeras to *T. canis* and *T. cati* TSOM, respectively, and the observed CLR binding range suggests a resemblance of antigens derived by these two species. MCL/TSOM interaction was most surprising as a contribution of MCL to helminth infection has not been shown to date. It is known that MCL dimerises with other CLRs such as Mincle and Dectin-2 and is involved in immunity against mycobacteria by recognising trehalose dimycolate (TDM, mycobacterial cord factor) [36,37]. Thus, potential involvement of MCL in toxocarosis was a novel finding.

In contrast to TSOM, MGL-1 was the only CLR prominently binding to both *T. canis*- and *T. cati*-derived TES, pointing towards an involvement of MGL-1 in recognition of TES in paratenic or definitive hosts. Human MGL and its murine orthologues MGL-1 and MGL-2 are known to recognise terminal *N*-acetylgalactosamine and galactose as well as Lewis X and A glycostructures and are frequently involved in recognition of helminths and their secreted antigens [38]. So far, MGL/parasite interactions were reported for *Schistosoma mansoni*, *Trichuris suis*, *Taenia crassiceps* and *Fasciola hepatica* [38,39]. Most interestingly, consistent with our results, Schabussova et al. (2007) revealed binding of a human MGL-hFc chimera to *T. canis* TES.

We were further able to define a distinct MGL-1-interacting area on the oral aperture of *T. canis* and *T. cati* L3. It has been previously shown that *T. canis* L3 possess carbohydrates located at this area, which seem to be immunogenic as they are highly reactive to antibodies directed against carbohydrate epitopes, that is, Tcn-4, Tcn-5 and Tcn-7, produced in mice upon infection [40]. Our findings suggest that these carbohydrate epitopes might represent the target structures for MGL-1. Interestingly, the mentioned carbohydrate epitope-directed antibodies bind to a 70 kDa fraction of *T. canis* TES as well as L3 surface glycoproteins [41]. This 70 kDa protein is also called TES-70 or Tc-CTL-4, which is a major component of TES and most likely present in oral areas of the L3 surface coat [40,42]. Tc-CTL-4 represents a PDCTL, which binds to the surface of canine epithelial cells and was the first TES compound reported to directly interact with mammalian cells [20,41]. In the present study, sodium dodecyl sulphate-polyacrylamide gel electrophoresis (SDS-PAGE) and subsequent immunoblot was used to evaluate whether CLR-hFc chimeras interacted with specific proteins or their glycostructures. Indeed, MGL-1 binding to a *T. canis*-derived protein fraction of nearly 70 kDa was observed in CLR-hFc immunoblot. Although we have not identified a distinct ligand yet, the size of the MGL-1-interacting protein fraction resembled that of Tc-CTL-4, which might represent a potential target for MGL-1. However, this has to be clarified in future studies. It is likely that MGL-1 binds to analogous *T. cati*-derived components as supposed by the close phylogenetic relationship and similarities between *T. canis* and *T. cati* L3 [43]. Moreover, MGL-1 interacted with the same oral area in both *T. canis*- and *T. cati* L3. Interestingly, we observed a substantial difference in composition of *T. cati* TSOM and TES antigens, with MGL-1 binding to a lower fraction of *T. cati*-derived antigens compared to those of *T. canis*. Only few protein sequences of the abundant TES antigens of *T. cati* are deposited at the NCBI, among them also Tc-CTL-4, which is only 83.6% identical to the same protein found in *T. canis* TES. So far, components of *T. cati*-derived surface and TES antigens have been insufficiently characterised [44,45].

MCL, on the other hand, did not show visible binding to parasite L3, suggesting an interaction with an (internal) ligand that is not accessible on the surface. This is supported by the fact that MCL revealed binding to TSOM in ELISA-based binding studies only. Interestingly, MCL displayed equal binding patterns compared to MGL-1, recognising a band of 65 kDa (*T. canis*) and 55 kDa (*T. cati*) in both TSOM and TES in immunoblot. However, SDS-PAGE was performed under denaturing conditions which affects folding conformation of proteins, thus potentially changing their binding properties to antibodies [46]. Another explanation might be that the MCL-binding site is masked in natural TES. Overall, *Toxocara*-derived ligands for MGL-1 and MCL have to be investigated in future studies, for instance by more precise analysis via mass spectrometry. Furthermore, MGL-1 and MCL might not only interact with glycoproteins but also other glycoconjugates, such as glycolipids, that are not covered by the immunoblot methodology carried out in the present study. Moreover, it would be interesting to know whether canine or feline CLRs might recognise TES and TSOM. Accordingly, orthologues of MGL-1 and MCL exist in both the domestic dog and cat, thus being potential CLRs for the recognition of *Toxocara*-derived antigens in the definitive host.

To gain first insights into the immunological relevance of CLR/*Toxocara* spp. interaction, BMDC stimulation assays were performed. In general, stimulation studies with antigens derived from helminths, including *Toxocara* spp., are challenging as these antigens seem to induce rather low levels of cytokine secretion and surface marker expression or even dampen effector functions of APCs [47]. This was also observed in other studies utilising canine monocyte-derived DCs or a human THP-1 macrophage cell line [23,48]. Thus, a co-stimulus, often LPS as a TLR4 agonist, inducing pro-inflammatory cytokine secretion is generally needed and was used for stimulation studies of WT and CLR^−/−^ DCs. Furthermore, migrating larvae lead to substantial damage and lesions in invaded tissues [49,50,51], thus contributing to cell death and the release of damage-associated molecular patterns (DAMPs), which contain high amounts of TLR4 agonists such as heat shock proteins and extracellular matrix molecules [52]. Thus, LPS represents a suitable co-stimulus in in vitro assays, mimicking endogenous stimuli released upon invasion of larvae. Overall, almost nothing is known about the mechanistic response of DCs to *Toxocara* spp. antigen stimulation. Junginger et al. [48] observed that *T. canis* TES prevented canine DCs from LPS-induced maturation as indicated by reduced MHC-II and CD86 surface marker expression. In the present study, TES stimulation had little to no effect on MHC-II surface marker expression of murine BMDCs. This might be explained by the difference in the origin of DCs (canine monocyte-derived DCs vs. murine BMDCs) and stimulation conditions (TES stimulation prior to LPS stimulation vs. simultaneous TES/LPS stimulation). To the best of our knowledge, this is the first report of a TSOM- and TES-mediated cytokine production by murine BMDCs, revealing a synergistic effect of LPS/TSOM and TES stimulation on IL-6 and TNF cytokine production. Strikingly, this effect was even more pronounced in MCL^−/−^ BMDCs, possibly indicating that MCL mediates downregulation of cytokine secretion or affects signalling pathways of other receptors, for example, the LPS-recognising receptor TLR4, upon *T. cati* stimulation. In this context, MCL was recently reported to interfere with TLR4 signalling in anti-tumour response [53]. Furthermore, MCL might also affect *Toxocara*-mediated Mincle and Dectin-2 signalling cascades as MCL is closely linked to expression and signalling of these CLRs [36,37,54].

Although we did not observe any difference in DC effector function upon MGL-1 deficiency, immunological relevance of this CLR should be further investigated in future studies. We might have missed a contribution of MGL-1 to *Toxocara*-mediated innate immunity in our initial stimulation studies as MGL-dependent immune response to other helminth infections is frequently reported. In particular, human MGL interacts with antigens of *S. mansoni* and *T. suis*, inhibiting the TLR-mediated activation of immature dendritic cells [55,56,57,58], whereas murine MGL-1 binds to ES antigens of *T. crassiceps*, thus dampening pro-inflammatory immune responses [59,60]. Furthermore, *F. hepatica* triggers anti-inflammatory immune responses through MGL, thereby suppressing innate and adaptive immunity by reduced Th1 polarisation [39]. Evaluation of additional DC effector functions or those of alternatively activated macrophages, which are highly relevant in helminth infections [61], could aid in identifying the immunological relevance of MGL-1/*Toxocara* spp. interactions.

## 4. Materials and Methods

### 4.1. Preparation of Toxocara spp. Antigens

Experimental *Toxocara* infection of dogs and cats was permitted by the ethics commission (Animal Care and Use Committee) of the German Lower Saxony State Office for Consumer Protection and Food Safety (*Niedersächsisches Landesamt für Verbraucherschutz und Lebensmittelsicherheit*) under reference number 33.9-42502-05-01A038. Eggs were purified from faeces of *T. canis*-infected dogs (field isolate HannoverTcanis2008) and *T. cati*-infected cats (field isolate HannoverTcati2010) by the sedimentation-flotation method, and allowed to embryonate in tap water for at least 6 weeks at room temperature (RT) with periodic oxygenation. Larval development was assessed each week and hatching was initiated if at least 90% of eggs were infective.

For egg hatching, the eggs’ outer surface layer was dissociated by incubation with 12% sodium hypochlorite solution (Carl Roth, CAT#9062.3, Karlsruhe, Germany) for 5 min. Eggs were washed 4–6 times with 50 mL phosphate-buffered saline (PBS) until pH 7.0 was reached. Following centrifugation at 1500 g for 5 min, eggs were transferred to cultivation medium [RPMI 1640 (PAN Biotech, CAT#P04-16516, Aidenbach, Germany) supplemented with 2 mM L-glutamine (PAN Biotech, CAT#P04-82050, Aidenbach, Germany), 100 U/mL penicillin/100 μg/mL streptomycin (PAN Biotech, CAT#P06-07025, Aidenbach, Germany) and 250 ng/mL amphotericin B (PAN Biotech, CAT#P06-01025, Aidenbach, Germany)]. Eggs were gassed with CO_2_ for 5 min and placed onto a 40 µm cell strainer (Sarstedt, CAT#83.3945.040, Nümbrecht, Germany) covered in cultivation medium in a 50 mL falcon to allow migration of larvae to the bottom of the tube for 48 h. The larval pellet was washed two times with 50 mL cultivation medium and transferred to a 12 well tissue culture plate (Sarstedt, CAT#83.3921, Nümbrecht, Germany) with 50,000 larvae per well. Every two to three days, the supernatant was harvested and exchanged. Obtained supernatant containing TES was sterile-filtered through a 0.2 µm syringe filter (Sarstedt, CAT#83.1826.001, Nümbrecht, Germany) and frozen at −80 °C until further use. After 2–4 weeks of cultivation, larvae were pooled and frozen for 24 h at −20 °C.

For production of TSOM, cultivated larvae were washed 4 times in 50 mL PBS, transferred to tubes containing 0.5 mm glass beads (Qiagen, CAT#13116-50, Hilden, Germany) and homogenised using the Precellys^®^ 24 homogeniser (VWR, CAT#432-3750, Darmstadt, Germany). The homogenate was centrifuged at 13,000 g for 10 min at 4 °C and the supernatant containing the soluble TSOM was aliquoted and frozen at −80 °C.

Protein concentrations of *T. canis* and *T. cati* TES and TSOM were measured by Pierce™ Detergent Compatible Bradford Assay (Thermo Scientific, CAT#23246, Waltham, MA, USA) according to the manufacturer´s micro microplate protocol with slight modifications. Briefly, 150 µL of antigen solution was mixed with 150 µL of assay reagent and incubated for 10 min. OD was measured in a Biowave 340 photometer (BioTek, Bad Friedrichshall, Germany) at a wavelength of 595 nm and protein concentration was calculated related to a bovine γ-globulin standard (Bio-Rad, CAT#5000208, Feldkirchen, Germany) ranging from 0 to 200 µg/mL. For confirmation and comparison of different antigen batches, 0.5 µg of each batch was separated via 10% SDS-PAGE and visualised by silver staining as previously described [62,63].

### 4.2. CLR-hFc Fusion Proteins for Detection of CLR/Toxocara spp. Interactions

CLR-hFc fusion proteins were generated as previously described [31,64]. Briefly, extracellular domain sequences of the respective CLRs were inserted into an expression vector containing the Fc part of human IgG1 (pFUSE-hIgG1-Fc2, Invivogen, CAT#pfuse-hg1fc2, San Diego, CA, USA) and CHO-S cells were transiently transfected with the CLR-hFc-expression vector using polyethylenimine (PEI, Polysciences, CAT#23966-2, Hirschberg an der Bergstrasse, Germany). CLR-hFc fusion proteins were purified from the supernatant by affinity chromatography via HiTrap protein G columns (GE Healthcare, CAT#17-0404-01, Chicago, IL, USA). SDS-PAGE with subsequent hFc-detecting immunoblot were applied to confirm the purity and identity of obtained fusion proteins. Modular design of these fusion proteins enabled the use of CLR-hFc chimeras for detection of CLR/*Toxocara* spp. interactions in ELISA-, fluorescence microscopy- and immunoblot-based methods.

### 4.3. CLR-hFc ELISA Binding Assays

For ELISA binding assays, medium binding half area 96 well microplates (Greiner Bio-One, CAT#675001, Cambridgeshire, UK) were coated with 8 µg/mL TSOM or TES of *T. canis* or *T. cati* at 4 °C overnight. After each step, the plates were washed three times with 0.05% Tween 20^®^ (Carl Roth, CAT#9127.1, Karlsruhe, Germany) in PBS and wells were blocked with 1% bovine serum albumin (BSA; Thermo Scientific, CAT#10398062, Waltham, MA, USA) in PBS for 2 h. CLR-hFc fusion proteins or hFc only (negative control) were added at a concentration of 5 µg/mL in lectin binding buffer [*n* = 4 in duplicates, 50 mM HEPES (Carl Roth, CAT#HN78.3, Karlsruhe, Germany), 5 mM CaCl_2_ (Carl Roth, CAT#CN93.1, Karlsruhe, Germany), 5 mM MgCl_2_ (Carl Roth, CAT#KK36.2, Karlsruhe, Germany), pH 7.4] or Ca^2+^-complexing EDTA buffer [*n* = 2 in duplicates, 50 mM HEPES (Carl Roth, CAT#HN78.3, Karlsruhe, Germany), 10 mM EDTA (Carl Roth, CAT#CN06.2, Karlsruhe, Germany) pH 7.4] and incubated for 1 h at RT. Bound fusion proteins were detected by a HRP-conjugated anti-human IgG (Fc) antibody (Jackson ImmunoResearch Labs, CAT#109-035-098, Cambridgeshire, UK) diluted 1:5000 in 1% BSA (Thermo Scientific, CAT#10398062, Waltham, MA, USA), 0.05% Tween 20^®^ (Carl Roth, CAT#9127.1, Karlsruhe, Germany) in PBS for 1 h at RT. Colorimetric development was achieved by addition of o-phenylenediamine dihydrochloride (OPD, Thermo Scientific, CAT#34006, Waltham, MA, USA) substrate solution and stopped with 2.5 M sulphuric acid (Carl Roth, CAT#4623.4, Karlsruhe, Germany). OD was measured at a wavelength of 495 nm in a Multiscan GO photometer (Thermo Scientific, CAT#N13135, Waltham, MA, USA). CLR binding was defined as an OD value exceeding four times the OD of averaged hFc negative controls.

### 4.4. Fluorescence Microscopy-Based Binding Assay to Toxocara Larvae

As the results of ELISA-based binding studies revealed the interaction of the MCL with helminth-derived antigens and distinct binding of MGL-1 to TES, the following experiments were performed with these two CLR-hFc fusion proteins. Freshly hatched *T. canis* and *T. cati* L3 were fixed with 1% paraformaldehyde (Carl Roth, CAT#0335.1, Karlsruhe, Germany) in PBS for 20 min at RT followed by incubation with respective CLR-hFc fusion proteins or hFc only (negative control) at a concentration of 10 µg/mL in Dulbecco’s Modified Eagle Medium (DMEM, PAN Biotech, CAT#P04-01548S1, Aidenbach, Germany) supplemented with 10% fetal bovine serum (FBS; PAN Biotech, CAT#P30-1302, Aidenbach, Germany) for 2 h at 4 °C. An anti-human IgG (Fc) AlexaFluor 488-labeled antibody (Jackson ImmunoResearch Labs, CAT#109-545-098, Cambridgeshire, UK) diluted 1:200 in 1% FBS (PAN Biotech, CAT#P30-1302, Aidenbach, Germany) in PBS was used for detection of bound CLR hFc-fusion proteins. After incubation for 2 h at 4 °C, larvae were embedded in DAPI-containing proLong™ Gold antifade mountant (Invitrogen, CAT#P10144, Waltham, MA, USA) and staining was visualised using an Axio Imager M2 microscope (Zeiss, CAT#Axio Imager.M2, Oberkochen, Germany).

### 4.5. CLR-hFc Recognition of Toxocara Antigen Fractions

For immunoblot-based binding studies, 2.0 µg TSOM and 0.2 µg TES of *T. canis* or *T. cati* as well as negative controls PBS (con_TSOM_) and medium (con_TES_) were separated by 10% denaturing SDS-PAGE and transferred to a nitrocellulose membrane (Macherey-Nagel, CAT#741280, Düren, Germany). The membrane was blocked with 5% milk powder (Carl Roth, CAT#T145.3, Karlsruhe, Germany) in TBS containing 0.05% Tween 20^®^ (Carl Roth, CAT#9127.1, Karlsruhe, Germany) for 1 h at RT with agitation followed by incubation with MGL-1 and MCL CLR-hFc fusion proteins or hFc only (negative control) at a concentration of 1 µg/mL in lectin binding buffer [50 mM HEPES (Carl Roth, CAT#HN78.3, Karlsruhe, Germany), 5 mM CaCl_2_ (Carl Roth, CAT#CN93.1, Karlsruhe, Germany), 5 mM MgCl_2_ (Carl Roth, CAT#KK36.2, Karlsruhe, Germany), pH 7.4] for 1 h at RT with agitation. For detection of bound fusion proteins, the membrane was incubated with HRP-conjugated anti human IgG (Fc) antibody (Jackson ImmunoResearch Labs, CAT#109-035-098, Cambridgeshire, UK) diluted 1:10,000 in TBS containing 0.05% Tween 20^®^ (Carl Roth, CAT#9127.1, Karlsruhe, Germany) for 1 h at RT with agitation. Chemiluminescence was induced by addition of Amersham ECL Western Blot detection reagent (GE Healthcare, CAT#RPN2232, Chicago, IL, USA) with subsequent detection in a Celvin^®^ S 320+ imager (BIOSTEP, CAT#31-56-204, Burkhardtsdorf, Germany).

### 4.6. Preparation of Bone Marrow Cells

The sacrifice of mice for scientific purposes was approved by the Animal Welfare Officer of the University of Veterinary Medicine Hannover, Germany (AZ 02.05.2016). MGL-1^−/−^ mice were generated as previously described [65]. Miyake et al. (2013) generated MCL^−/−^ mice, which were obtained from the National Institutes of Health-sponsored Mutant Mouse Regional Resource Center (MMRRC) National System as described previously [66]. MGL-1^−/−^, MCL^−/−^ and C57BL/6J WT control mice (own breeding) were maintained under specific pathogen-free conditions in individual ventilated cages (Tecniplast Sealsafe Green Line, 391 × 199 × 160 mm) at a 12:12 h light-dark cycle with food and water provided *ad libitum*. Bone marrow cells were obtained from the femur and tibia of MGL-1^−/−^, MCL^−/−^ and WT mice.

### 4.7. MGL-1^−/−^ and MCL^−/−^ BMDC Stimulation Assays

DCs play a predominant role in differentiation and priming of T cells, thereby shaping adaptive immune responses which are crucial in *Toxocara* immunity [6,67]. Thus, we performed BMDC stimulation assays to evaluate the effect of MGL-1 and MCL/*Toxocara* interaction on BMDC effector functions. To this end, WT and MGL-1^−/−^ and MCL^−/−^ bone marrow cells were differentiated into BMDCs using BMDC differentiation medium [IMDM medium (PAN Biotech, CAT#P04-20250, Aidenbach, Germany) supplemented with 10% FBS (PAN Biotech, CAT#P30-1302, Aidenbach, Germany), 2 mM L-glutamine (PAN Biotech, CAT#P04-82100, Aidenbach, Germany), 100 U/mL penicillin/100 μg/mL streptomycin (PAN Biotech, CAT#P06-07100, Aidenbach, Germany), 10% X63-GM-CSF supernatant] at 37 °C and 5% CO_2_. After 9 days of differentiation, BMDCs were seeded at a concentration of 5 × 10^5^ cells/mL in a 96-well plate and stimulated with LPS (1 ng/mL, Sigma-Aldrich, CAT#tlrl-3pelps, St. Louis, MO, USA) as positive control (MGL-1^−/−^: *n* = 2 in duplicates, MCL^−/−^: *n* = 3 in duplicates). It was shown that helminth products often fail to induce direct activation of DCs or even suppress DC maturation [47] and it can be assumed that tissue-invading larvae cause substantial damage, thereby inducing the cell death-mediated release of TLR agonist-containing DAMPs [49,52]. Thus, BMDC stimulations with *T. canis* (MGL-1^−/−^: *n* = 2 in duplicates, MCL^−/−^: *n* = 3 in duplicates) and *T. cati* (MGL-1^−/−^: *n* = 2 in duplicates, MCL^−/−^: *n* = 3 in duplicates) TSOM (2.5, 25 and 50 µg/mL) or TES (2.5 µg/mL) were simultaneously pulsed with LPS (1 ng/mL) at 37 °C and 5% CO_2_. After 22 h, supernatants were harvested and concentrations of the cytokines IL-6 and TNF measured by ELISA (murine IL-6 ELISA Kit, R&D, CAT#DY-406-05, Minneapolis, MN, USA; murine TNF-α ELISA Kit, R&D, CAT#DY-410-05, Minneapolis, MN, USA). BMDCs were blocked with anti-mouse CD16/32 [93, eBioscience, CAT#14-0161-86, Waltham, MA, USA; diluted 1:100 in staining buffer (PBS supplemented with 1% FBS, PAN Biotech, CAT#P30-1302, Aidenbach, Germany)] for 10 min at 4 °C. BMDC differentiation was verified by staining with APC-conjugated anti-mouse CD11c (N418, eBioscience, CAT#17-0114-82, Waltham, MA, USA; diluted 1:200 in staining buffer). Antigen presenting properties were evaluated by staining the surface markers MHC-II with FITC-conjugated anti-mouse MHC-II (AF6-120.1, BD Pharmingen, CAT#553551, San Jose, CA, USA; diluted 1:100 in staining buffer) and CD86 with PE-conjugated anti-mouse CD86 (B7-2, eBioscience, CAT#12-0862-83, Waltham, MA, USA; diluted 1:200 in staining buffer) for 20 min at 4 °C. Samples were analysed using an Attune NxT Flow Cytometer (Thermo Fisher Scientific, CAT#A28993, Waltham, MA, USA).

### 4.8. Statistics

All analyses were performed using GraphPad Prism (version 8, GraphPad Software, La Jolla, CA, USA). CLR-hFc ELISA binding assays and MGL-1^−/−^ BMDC stimulation assays were analysed by paired, and MCL^−/−^ BMDC stimulation assays by unpaired Student’s *t*-test. In all analyses, a *p*-value ≤ 0.05 was considered statistically significant.

## 5. Conclusions

The present study shows the binding of various CRLs to *Toxocara* TSOM, distinct binding to TES antigens, and reveals two promising candidate CLRs for immune modulation during *Toxocara* infection: MGL-1 and MCL. MGL-1 prominently interacted with *T. canis* and *T. cati* L3 and their TES products as indicated by binding of the MGL-1-hFc chimera to oral surface structures, total TES and protein fractions. *Toxocara*/MCL interaction represents a new discovery. So far, this CLR was only shown to recognise bacterial agents. In addition, MCL interfered with *T. cati* antigen-mediated stimulation, thus leading to higher cytokine secretion in MCL^−/−^ compared to WT BMDCs. In summary, the present study allows insights into the recognition of *Toxocara* spp. by CLRs and suggests a potential role in *Toxocara*-mediated immune modulation. Future studies should investigate distinct ligands recognised by MGL-1 as well as MCL and characterise the detailed immunological relevance of CLR/*Toxocara* spp. interaction, for instance by challenging WT and CLR^−/−^ mice with infective *T. canis* and *T. cati* L3.

## Data Availability

The original contributions presented in the study are included in the article/supplementary material, further inquiries can be directed to the corresponding author.

## Supplementary Materials

The following are available online at https://www.mdpi.com/2076-0817/10/3/321/s1, Figure S1: MGL-1 binds to the remaining sheath of *T. canis* second stage larva, Table S1: Statistical analysis of *Toxocara* spp. antigens binding to CLR-hFc fusion proteins in ELISA-based studies, Table S2: Statistical analysis of *Toxocara* spp.-antigens mediating elevated cytokine secretion of WT and MCL^−/−^ bone marrow-derived dendritic cells (BMDCs).
