# Supplementary material for: Toxocara canis and Toxocara cati Somatic and Excretory-Secretory Antigens Are Recognised by C-Type Lectin Receptors
Source: Pathogens. 2021 Mar 9;10(3):321. doi: 10.3390/pathogens10030321 (PMC8001263; doi:10.3390/pathogens10030321)
Supplement: Supplementary file 1 [file pathogens-10-00321-s001.pdf]

**Supplementary Material**

# *Toxocara canis* and *Toxocara cati* somatic and excretory-secretory antigens are recognised by C-type lectin receptors

Marie-Kristin Raulf, Bernd Lepenies, Christina Strube

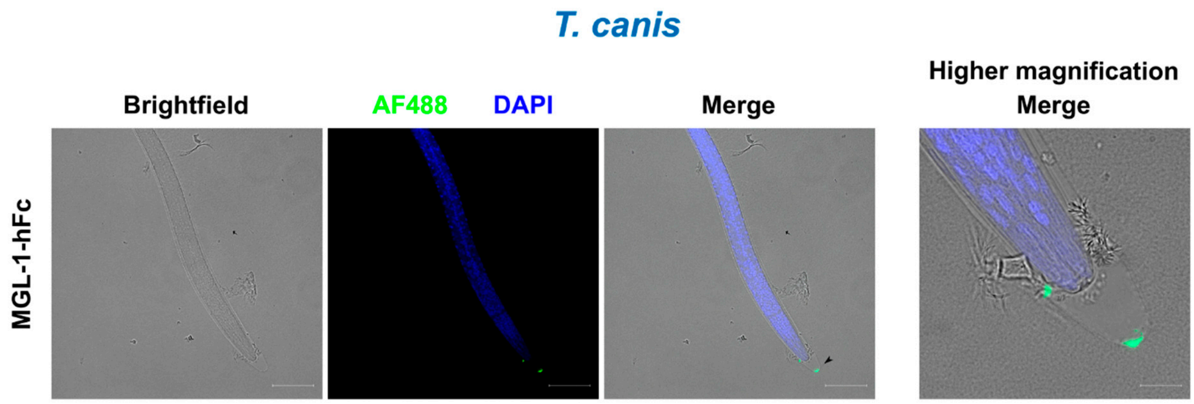


**Figure S1. MGL-1 binds to the remaining sheath of *T. canis* second stage larva.** Scale bar represents 50 µm for lower magnification (left images) and 10 µm for higher magnification (right image). Green fluorescence: CLR-hFc fusion protein, blue fluorescence: DAPI-stained DNA.

**Table S1. Statistical analysis of *Toxocara* spp. antigens binding to CLR-hFc fusion proteins in ELISA-based studies.** The table shows *P*-values of respective *Toxocara*/CLR binding compared to *Toxocara*/hFc binding (negative control) by paired, two-tailed Student's *t*-test. Results were considered statistically significant if $P \leq 0.05$ (printed in bold). TSOM: *Toxocara* somatic antigen, TES: *Toxocara* excretory-secretory antigen, EDTA: ethylenediamine tetraacetic acid (test for $Ca^{2+}$ dependency), n.d.: not determined, *: mean OD values exceeding the threshold of 0.24.

| | ***T. canis*** | | | | ***T. cati*** | | | |
|---|---|---|---|---|---|---|---|---|
| hFc vs. | TSOM | TES | TSOM/ EDTA | TES/ EDTA | TSOM | TES | TSOM/ EDTA | TES/ EDTA |
| Mincle | **≤0.001*** | **0.004*** | **0.038*** | **0.034** | **0.001*** | **0.012** | 0.051 | 0.110 |
| Dectin-1 | **0.002*** | **0.004*** | **0.007*** | **0.015** | **0.002*** | **0.004** | 0.102* | 0.100 |
| Dectin-2 | **0.019*** | **≤0.001** | 0.118* | **≤0.001*** | **0.030*** | **0.032*** | 0.056* | 0.147* |
| SIGNR3 | **0.011** | **0.011** | **0.033** | 0.087 | **0.028*** | **0.013** | 0.107 | 0.198 |
| DC-SIGN | **0.007*** | **0.006*** | 0.061 | 0.078 | **0.009*** | **0.023*** | **0.048** | 0.136 |
| CLEC12A | **0.006*** | **0.003** | **0.027*** | 0.077 | **0.021*** | **0.004** | 0.089* | 0.086 |
| CLEC12B | **0.015*** | 0.053 | 0.086 | 0.135 | **0.006*** | **0.041** | 0.113 | 0.070 |
| MDL-1 | 0.110 | 0.070 | 0.337 | 0.230 | 0.164 | 0.110 | 0.283 | 0.143 |
| MGL-1 | **0.002*** | **≤0.001*** | 0.118 | **0.050** | **0.003*** | **0.001*** | 0.165 | 0.209 |
| MCL | **0.011*** | 0.051 | 0.299 | 0.337 | **0.025*** | 0.071 | 0.500 | 0.178 |
| Langerin | **0.001*** | **0.047** | **0.034*** | 0.241 | **≤0.001*** | **0.001** | **0.008*** | 0.166 |

**Table S2. Statistical analysis of *Toxocara* spp.-antigens mediating elevated cytokine secretion of WT and MCL$^{-/-}$ bone marrow-derived dendritic cells (BMDCs).** The table shows *P*-values of respective *Toxocara* antigen stimulation compared to LPS stimulation by unpaired, two-tailed Student's *t*-test. Results were considered statistically significant if $P \leq 0.05$ (printed in bold). TSOM: *Toxocara* somatic antigens, TES: *Toxocara* excretory-secretory products.

| | IL-6 | | | | TNF-α | | | |
|---|---|---|---|---|---|---|---|---|
| | *T. canis* | | *T. cati* | | *T. canis* | | *T. cati* | |
| LPS vs. | WT | MCL$^{-/-}$ | WT | MCL$^{-/-}$ | WT | MCL$^{-/-}$ | WT | MCL$^{-/-}$ |
| unstimulated | **≤0.001** | **≤0.001** | **≤0.001** | **≤0.001** | **0.001** | **≤0.001** | **0.001** | **≤0.001** |
| TSOM 2.5 µg/ml | 0.642 | **0.007** | 0.175 | **≤0.001** | 0.737 | **0.017** | 0.547 | **0.001** |
| TSOM 25 µg/ml | **0.002** | **≤0.001** | **0.001** | **≤0.001** | **0.004** | **0.003** | **0.003** | **≤0.001** |
| TSOM 50 µg/ml | **0.004** | **0.007** | **≤0.001** | **≤0.001** | **0.005** | **0.042** | **≤0.001** | **≤0.001** |
| TES 2.5 µg/ml | **0.006** | 0.534 | **≤0.001** | **≤0.001** | **0.001** | **0.033** | **≤0.001** | **≤0.001** |
